# Description of *Seinura italiensis* n. sp. (Tylenchomorpha: Aphelenchoididae) found in the medium soil imported from Italy

**DOI:** 10.21307/jofnem-2020-018

**Published:** 2020-03-18

**Authors:** Jianfeng Gu, Munawar Maria, Lele Liu, Majid Pedram

**Affiliations:** 1 Technical Centre of Ningbo Customs (Ningbo Inspection and Quarantine Science Technology Academy), No. 8 Huikang, Ningbo, 315100, Zhejiang, P.R. China; 2 Laboratory of Plant Nematology, Institute of Biotechnology, College of Agriculture and Biotechnology, Zhejiang University, Hangzhou, 310058, Zhejiang, P.R. China; 3 Department of Plant Pathology, Faculty of Agriculture, Tarbiat Modares University, Tehran, Iran

**Keywords:** Molecular, Morphology, Phylogeny, Predatory, Taxonomy

## Abstract

*Seinura italiensis* n. sp. isolated from the medium soil imported from Italy is described and illustrated using morphological and molecular data. The new species is characterized by having short body (477 (407-565) μm and 522 (469-590) μm for males and females, respectively), three lateral lines, stylet lacking swellings at the base, and excretory pore at the base or slightly anterior to base of metacorpus; females have 58.8 (51.1-69.3) μm long post-uterine sac (PUS), elongate conical tail with its anterior half conoid, dorsally convex, and ventrally slightly concave and the posterior half elongated, narrower, with finely rounded to pointed tip and males having seven caudal papillae and 14.1 (12.6-15.0) μm long spicules. Morphologically, the new species is similar to *S. caverna*, *S. chertkovi*, *S. christiei*, *S. hyrcania*, *S. longicaudata*, *S. persica*, *S. steineri*, and *S. tenuicaudata*. The differences of the new species with aforementioned species are discussed. In molecular phylogenetic analyses using near full-length small and large subunit ribosomal DNA (SSU and LSU rDNA D2-D3 expansion segments) sequences, the new species fell into a clade including three previously described/sequenced species of the genus in both SSU and LSU Bayesian phylogenetic trees.

*Seinura* (Fuchs, 1931) is a member of the family Aphelenchoididae (Skarbilovich, 1947) and is known for its predatory behavior on other nematode species. The word *Seinura* is derived from *seios* meaning move to and fro and *oura* meaning tail (Hunt, 1993). The genus contains over 50 species distributed across different climatic zones and environments (Kaisa, 2000; Bajaj, 2015; Adeldoost et al., 2016; Kanzaki et al., 2018). As *Seinura* species are not qualified as pest species, this group has not receive special attention in China. The outbreak of pine wilt disease accelerated the research on the aphelenchs in China, and several nationwide surveys were conducted on the pine trees that resulted in the documentation of *Seinura* species as well. Till now, *S. aurangabadensis* (Suryawanshi, 1971), *S. elmiraensis* (van der Linde, 1938; Goodey, 1960), *S. filicaudata* (Christie, 1939; Goodey, 1960), *S. oahueensis* (Christie, 1939; Goodey, 1960), *S. oostenbrinki* (Husain and Khan, 1967), *S. steineri* (Hechler, 1965 in Hechler and Taylor, 1965), *S. tenuicaudata* (de Man, 1895; Goodey, 1960), and *S. tritica* (Bajaj and Bhatti, 1982) have been documented from China (Jiang, 2000; Xie and Zhang, 2003; Huang and Ye, 2008; Feng, 2010; Ding et al., 2013; Zhang et al., 2013). The two previously described species *S. lii* (Huang and Ye, 2006) and *S. wuae* (Huang and Ye, 2006) from China are now transferred to *Aphelenchoides* (Fischer, 1894) and *Bursaphelenchus* (Fuchs, 1937), respectively (Gu et al., 2017; Kanzaki et al., 2018).

The present study describes a new *Seinura* species isolated from medium soil of imported *Olea europaea* L. from Italy. The species was compared with all related species and found to be a new member of the genus, being described herein as *Seinura italiensis* n. sp.

## Materials and methods

### Nematode isolation and morphological study

Medium soil collected from imported *Olea europaea* from Italy to Ningbo, China, was sent to the nematology laboratory for nematode detection. The nematodes were isolated by the modified Baermann funnel technique for 24 hr. Permanent slides were prepared by heat-killed and fixed nematodes with FA 4:1 and ethanol-glycerin dehydration according to Seinhorst (1959) as modified by De Grisse (1969). Morphometrics, drawings, and light micrographs of nematodes were done with the aid of a Zeiss microscope equipped with a Zeiss AxioCam MRm CCD camera.

### Molecular and phylogenetic analyses

DNA samples were prepared according to Li et al. (2008). Three sets of primers (synthesized by Majorbio, Shanghai, China) were used in the PCR analyses to amplify the near full-length SSU and D2-D3 expansion segments of LSU rDNA. The SSU region was amplified as two partially overlapping fragments; for the first fragment, the forward 988F (5′-CTC AAA GAT TAA GCC ATG C-3′) and reverse 1912R (5′-TTT ACG GTC AGA ACT AGG G-3′) primers were used and for the second part, the forward 1813F (5′-CTG CGT GAG AGG TGA AAT-3′) and reverse 2646R (5′-GCT ACC TTG TTA CGA CTT TT-3′) primers were used (Holterman et al., 2006). The LSU D2-D3 expansion segments were amplified with the forward primer D2A (5′-ACA AGT ACC GTG AGG GAA AGT TG-3′) and the reverse primer D3B (5′-TCG GAA GGA ACC AGC TAC TA-3′) (De Ley et al., 1999). PCR conditions were as described by Li et al. (2008) and Ye et al. (2007). PCR products were separated on 1.5% agarose gel and visualized by staining with ethidium bromide. PCR products of sufficiently high quality were sent for sequencing by Invitrogen, Shanghai, China.

The newly generated SSU and LSU sequences of *Seinura italiensis* n. sp. (accession numbers MN428135 and MN428136, respectively) were compared with those of other aphelenchoidid species available in GenBank using the BLAST homology search program. For reconstruction of SSU and LSU rDNA phylogenies, the homologous sequences of ektaphelenchid and seinurid species were retrieved from the database. The outgroup taxa were selected according to previous studies (Aliramaji et al., 2018, 2019). The selected sequences of both data sets were aligned using Clustal X2 (http://www.clustal.org/) with the default parameters. The editing of the resultant alignment was performed using MEGA (Tamura et al., 2011). The model of base substitution was selected using MrModeltest 2 (Nylander, 2004). The Akaike-supported model, a general time reversible model, including among-site rate heterogeneity and estimates of invariant sites (GTR+G+I) was used for both SSU and LSU analyses. Bayesian analyses were performed using MrBayes v3.1.2 (Ronquist and Huelsenbeck, 2003) with a random starting tree and running the chains for 5 × 10^6^ generations for both data sets. After discarding burn-in samples, the remaining samples were retained for further analyses. The Markov chain Monte Carlo (MCMC) method within a Bayesian framework was used to estimate the posterior probabilities of the phylogenetic trees (Larget and Simon, 1999) using the 50% majority rule. The convergence of model parameters and topology were assessed based on the average standard deviation of split frequencies and potential scale reduction factor values. The adequacy of the posterior sample size was evaluated using autocorrelation statistics as implemented in Tracer v.1.5 (Rambaut and Drummond, 2009). The output ﬁles of the phylogenetic trees were visualized using Dendroscope V.3.2.8 (Huson and Scornavacca, 2012) and re-drawn in CorelDRAW software version 2017. The Bayesian posterior probabilities (BPP) exceeding 0.50 are given on appropriate clades.

## Results

### Systematics

#### *Seinura italiensis* n. sp.

(Figs 1, 2).

**Figure 1:**
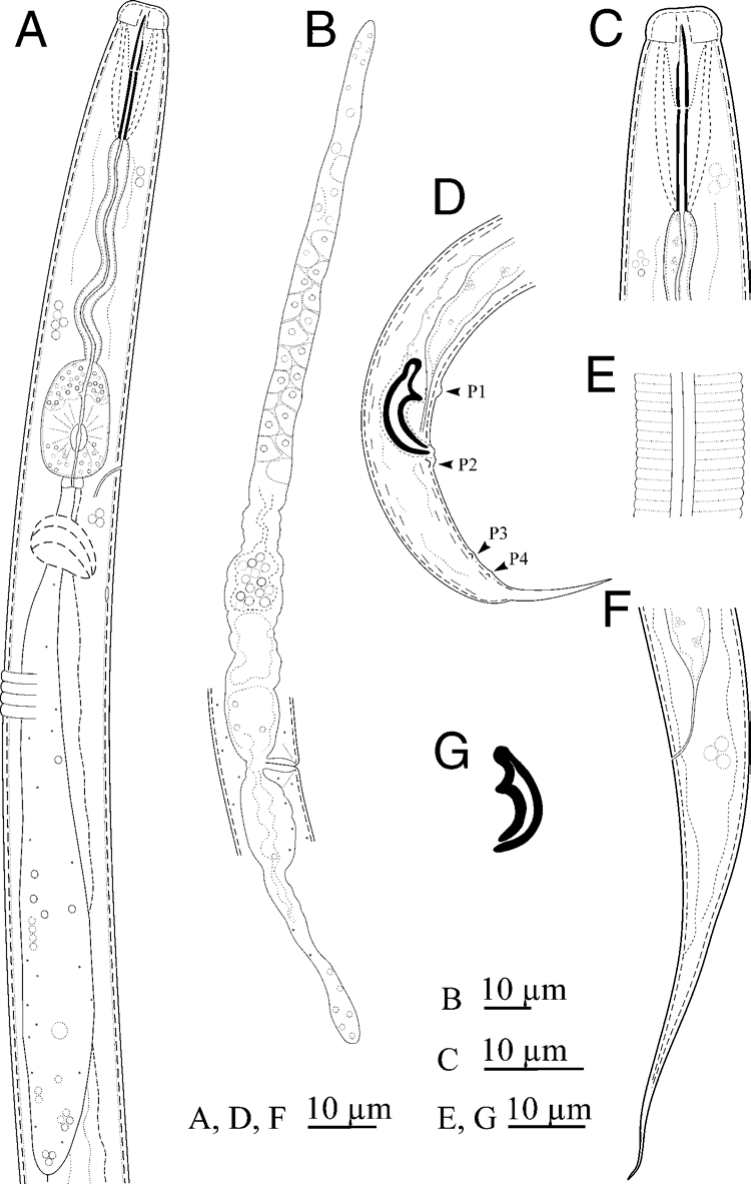
Line drawings of *Seinura italiensis* n. sp. (A): pharynx; (B): female reproductive system; (C): anterior region; (D): male posterior body region (arrows showing the P1-P4 papillae); (E): lateral lines; (F): female tail; (G): spicule. (Scale bars = A – G = 10 μm).

**Figure 2:**
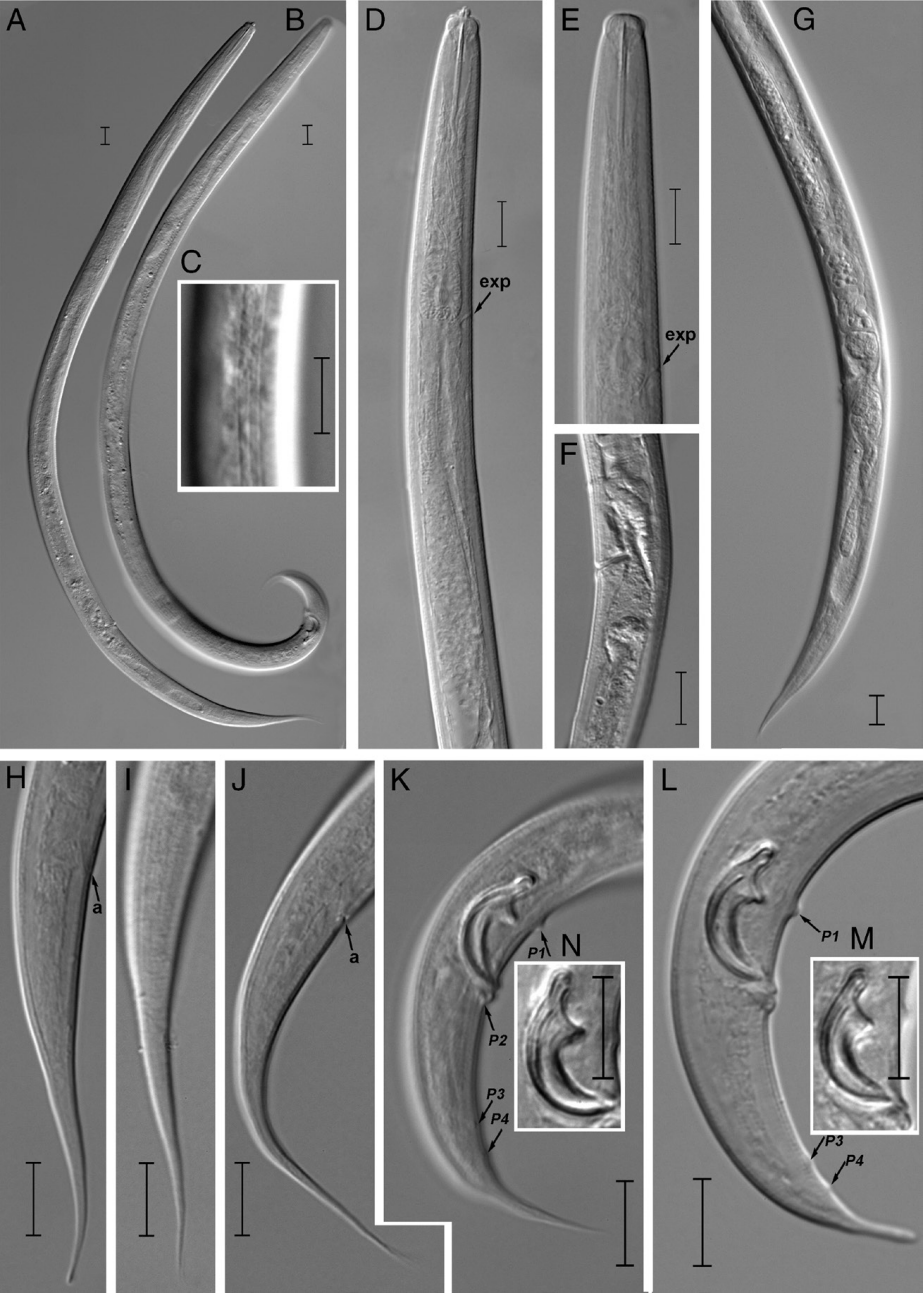
Light photomicrographs of *Seinura italiensis* n. sp. (A): entire female; (B): entire male; (C): lateral lines; (D, E): anterior region (arrows pointing on position of excretory pore); (F): vulval region; (G): female posterior region showing vulva and post-uterine sac; (H-J): female tail; (K, L): male tail (arrows showing position of caudal papillae); (M, N): spicules (Scale bars = A, B = 20 μm; C-N = 10 μm). exp = excretory pore; a = anus; P1 + P2 + P3 + P4 = caudal papillae.

### Measurements

Measurements of the new species are given in Table 1.

**Table 1.** Morphometrics of *Seinura italiensis* n. sp.

	Female		Male
Character	Holotype	Paratypes	Paratypes
n	–	15	15
L	478	522 ± 36.3 (469 – 590)	477 ± 41 (407 – 565)
a	30.1	29.6 ± 1.6 (26.7 – 33.7)	31.4 ± 2.1 (28.6 – 36.3)
b	6.6	7.0 ± 0.4 (6.3 – 7.5)	6.7 ± 0.5 (5.9 – 7.6)
b′	2.8	2.9 ± 0.2 (2.5 – 3.1)	3.1 ± 0.2 (2.8 – 3.5)
c	9.0	9.1 ± 1.3 (7.5 – 12.5)	12.5 ± 1.2 (10.5 – 14.6)
c′	5.3	5.6 ± 0.7 (3.9 – 6.5)	3.6 ± 0.3 (3.1 – 4.2)
V or T	73.4	72.2 ± 1.5 (69.7 – 75.3)	43.2 ± 11.2 (28.1 – 73.9)
Lip region height	3.6	3.4 ± 0.4 (2.8 – 4.1)	3.0 ± 0.3 (2.5 – 3.6)
Lip region width	7.3	7.3 ± 0.4 (6.6 – 8.1)	6.8 ± 0.6 (5.8 – 8.0)
Stylet length	19.8	20.6 ± 1.6 (18.3 – 23.6)	17.4 ± 1.5 (14.5 – 20.1)
Body diam.	15.9	17.7 ± 1.4 (15.7 – 20.9)	15.2 ± 1.2 (13.3 – 16.9)
Median bulb width	9.6	10.2 ± 0.6 (9.4 – 11.8)	8.7 ± 0.7 (7.5 – 10.2)
Median bulb length	16.4	17.8 ± 1.5 (14.8 – 21.0)	15.4 ± 1.0 (13.8 – 17.4)
Median bulb length/diam. ratio	1.7	1.7 ± 0.1 (1.5 – 1.9)	1.8 ± 0.1 (1.6 – 2.0)
Excretory pore from anterior end	61.9	67.9 ± 4.9 (57.3 – 74.4)	65.5 ± 3.8 (55.2 – 69.1)
Ovary or testis length	175.6	180.2 ± 35.8 (113 – 232)	206.0 ± 54.5 (143 – 340)
Post-uterine sac	59.2	58.8 ± 5.6 (51.1 – 69.3)	–
Vulva to anus distance	85.1	86.6 ± 7.4 (74.0 – 95.5)	–
Post-uterine sac length/vulva to anus (%)	69.6	68.2 ± 7.7 (58.1 – 85.3)	–
Anal (cloacal) body diameter	10.1	10.5 ± 0.7 (9.6 – 12.4)	10.7 ± 0.6 (9.6 – 11.6)
Tail length	53.1	58.3 ± 6.8 (43.6 – 72.0)	38.5 ± 4.5 (29.3 – 45.2)
Spicules (curved median line)	–	–	14.5 ± 1.0 (12.7 – 15.8)
Spicules (chord)	–	–	14.1 ± 0.8 (12.6 – 15.0)
Hemizonid from anterior end	90.7	84.4 ± 4.0 (77.6 – 91.8)	81.9 ± 4.5 (73.1 – 89.7)
Pharyngo-intestinal junction from anterior end	72.7	74.8 ± 3.6 (68.3 – 80.8)	71.0 ± 3.6 (63.5 – 76.0)
Pharyngeal gland from anterior end	170.7	182.1 ± 15.8 (156.8 – 222.2)	154.1 ± 10.6 (132.5 – 175.2)

Notes: All measurements are in μm and in the form: mean±sd (range).

## Female

Body cylindrical and ventrally arcuate, forming an open ‘C’ when heat relaxed. Cuticle distinctly annulated, with three incisures in lateral field. Cephalic (lip) region slightly offset, lip region *ca* twice as broad as high, lip sectors six, equally sized. Stylet comprises a conus *ca* 40% of total stylet length and a shaft without basal swellings. Stylet retractor muscles not clearly visible. Conophore absent. Procorpus cylindrical, metacorpus (median bulb) oval with glandular part occupying the one-third of anterior region (probably for storing pharyngeal secretions before injecting to the prey), and its valves situated post-centrally. The orifice of dorsal pharyngeal gland leads into lumen of metacorpus, *ca* one metacorpal valve length anterior to metacorpal valve. The pharyngeal glands form a long dorsal overlapping for *ca* 5-6 stylet length. There should be three nuclei, but only the posterior one is visible. Pharyngo-intestinal junction immediately posterior to the base of metacorpus. Nerve ring 3-4 metacarpal valve length posterior to metacorpus. Excretory pore at the base or slightly anterior to base of metacorpus. Hemizonid distinct in permanently mounted material, *ca* one metacorpus length posterior to excretory pore. Reproductive tract consists of an ovary, oviduct, spermatheca, crustaformeria, uterus, vagina + vulva, and PUS. Ovary single, outstretched, located on the right side of intestine, developing oocytes arranged in two rows, oviduct tube like, spermatheca rounded, *ca* one vulval body diam. long, filled with sperm in some individuals, crustaformeria inconspicuous, constructed of relatively large rounded cells, uterus with thick wall, vagina slightly inclined anteriorly, not sclerotized, the junction of uterus, PUS and vagina usually closed with no special structure such as a pair of three-celled structures found in several other aphelenchoidids, and vulva a simple slit in ventral view, without vulval membranes in lateral view, its anterior and posterior lips slightly raised. PUS *ca* 2-4 vulval body diam. long, extending for *ca* 58–85% of vulval-anus distance, sometimes filled with large sperm. Anus distinct, a dome-shaped slit in ventral view. Tail 4-7 anal body diam. long, its anterior half conoid, dorsally convex, ventrally slightly concave, the posterior half forming an elongate narrower section with finely rounded to pointed tip.

## Male

Body cylindrical and ventrally arcuate, forming a ‘J’ shape when heat relaxed. Cuticle and anterior body region similar to those of female. Gonad located on the right side of intestine and outstretched, with the anterior part of testis containing developing spermatocytes, larger spermatocytes arranged in remainder section of testis. Spicules mitten-shaped in lateral view, paired, and separate. Condylus broad, squarish, rostrum triangular with blunt tip directed ventrally. Condylus and rostrum form a well-developed capitulum with concave depression in middle, and calomus smoothly tapering together with lamina toward distil end. Gubernaculum or apophysis absent. Tail ventrally arcuate, tapering smoothly but narrowing at middle and distal half, forming a spike-like projection, with sharply pointed or bluntly rounded terminus. Bursal flap absent. Seven conspicuous genital papillae present (all papilliform), the single precloacal subventral papilla (P1) located at the level of rostrum, 8 to 10 μm anterior to the cloacal slit, the first subventral pair (P2) located at the level of cloacal slit (adcloacal), the second subventral pair (P3) located at *ca* two-thirds of tail length from cloacal slit, and the third ventral pair (P4) located at 3 to 4 μm posterior to P3.

## Type host and locality

The type material was isolated from medium soil of *Olea europaea* imported from to Ningbo, China on April 2018.

## Type specimens

The holotype female, 11 female and 8 male paratypes (slide numbers 2670-1 to 2670-15) were deposited in the nematode collection of Ningbo Customs Technical Centre, China. Four paratype females and seven paratype males (slide number T567) were deposited in the Canadian National Collection of Nematodes, Ottawa, Canada.

## Etymology

The species epithet is formed from the country of origin.

## Differential diagnosis

*Seinura italiensis* n. sp. is characterized by its short body (477 (407-565) μm and 522 (469-590) μm for males and females), a stylet lacking swellings at the base, and excretory pore located at the base of metacorpus or slightly anterior to its base, hemizonid *ca* one metacorpus length posterior to excretory pore; females with a 58.8 (51.1-69.3) μm long PUS and elongate conoid tail, and males with seven caudal papillae.

Based on the combination of (i) excretory pore at the base of metacarpus or slightly anterior, (ii) long PUS occupying over half of the vulva-anus distance; and (iii) elongate conoid tail with narrower distal region, the new species is similar to the following species of the genus: *S. caverna* (Kanzaki et al., 2018), *S. chertkovi* (Dmitrenko, 1966), *S. christiei* (Goodey, 1960), *S. hyrcania* (Adeldoost et al., 2016), *S. longicaudata* (Cobb, 1893; Goodey, 1960), *S. persica* (Adeldoost et al., 2016), *S. steineri* (Hechler and Taylor, 1965), and *S. tenuicaudata* (de Man, 1895; Goodey, 1960). The detailed comparisons of the new species with aforementioned species are as follows.

The new species can be differentiated from *S. caverna* by nature of its reproduction (amphimictic vs hermaphroditism), shorter female (522 (469-590) vs 778 (724-821) μm) and male body (477 (407-565) vs 722 μm), excretory pore position (67.9 (57.3-74.4) vs 77 (71-84) μm distance from anterior end in female), shorter female tail (58.3 (43.6-72.0) vs 112 (97-124) μm), shorter spicules (14.1 (12.6-15.0) μm vs 17.8 μm) and seven (vs six) male papillae; from *S. chertkovi* by shorter female body (522 (469-590) vs 615-700 μm), smaller a (29.6 (26.7-33.7) vs 35-41), b (7.0 (6.3-7.5) vs 8.2-9.0) and c′ (5.6 (3.9-6.5) vs 8.0) indices of female, longer spicules (14.1 (12.6-15.0) vs 10 μm) and number of genital papillae (seven vs eight); from *S. christiei* by shorter female (522 (469-590) vs 930-1,000 μm) and male body (477 (407-565) vs 650-720 μm), smaller a (29.6 (26.7-33.7) vs 31-39), b (7.0 (6.3-7.5) vs 10.0-11.2) and c′ (5.6 (3.9-6.5) vs 7) indices of female, shorter spicules (14.1 (12.6-15) vs 18-20 μm) and number of genital papillae (seven vs eight); from *S. hyrcania* by position of excretory pore (at the base of metacarpus or slightly anterior to it vs anterior to metacarpal valve), tail morphology (elongate conoid with narrower distal region vs very narrower distal end), length of PUS (58.8 (51.1-69.3) vs 13-23 μm, or 68.2 (58.1-85.3)% of vulva-anus distance vs 13-30%) and having (vs lacking) male; from *S. longicaudata* by shorter female body (522 (469-590) vs 800-1,700 μm), c (9.1 (7.5-12.5) vs 3.3-3.5) and c′ (5.6 (3.9-6.5) vs >20) indices of female; from *S. persica* by lateral lines (three vs four), longer (58.3 (43.6-72.0) vs 46 (36-55) μm) and differently shaped female tail (elongate conoid, dorsally convex, and ventrally slightly concave at anterior half, narrowing to an elongate section with finely rounded to pointed tip at distal half vs elongate conical, curved ventrally, attenuated asymmetrically, usually with a shallow depression dorsally, ending in a sharply pointed tip); from *S. steineri* by shorter female (522 (469-590) vs 790 (680-980) μm) and male body (477 (407-565) vs 517-715 μm), smaller b ratio of female (7.0 (6.3-7.5) vs 10.4 (8.5-11.8)), length of PUS (58.8 (51.1-69.3) vs 25-50 μm, or 68.2 (58.1-85.3)% of vulva-anus distance vs 40-50%) and c′ ratio of male (3.6 (3.1-4.2) vs 5); and from *S. tenuicaudata* by tail morphology (elongate conoid with narrower distal region vs elongate conoid with a hairy filiform distal section), shorter body of female (522 (469-590) vs 950 μm) and male (477 (407-565) vs 800 μm), smaller a (29.6 (26.7-33.7) vs 35-36), b (7.0 (6.3-7.5) vs 9.0-9.5) and c′ (5.6 (3.9-6.5) vs 9.8) indices of female, smaller b ratio of male (6.7 (5.9-7.6) vs 8.5-9.0) and shorter spicules (14.1 (12.6-15.0) μm vs 18 μm).

## Molecular profiles and phylogenetic status

The amplification and sequencing of near full-length SSU and LSU rDNA D2-D3 expansion segments of *Seinura italiensis* n. sp. yielded two single fragments of 1732 and 801 nucleotides long. The BLAST search using the SSU sequence revealed that it has 96.92% identity with the SSU rDNA of *S. caverna* (LC414971). The identity value of other sequences that showed high coverage was all less than 93%. The BLAST search using the LSU D2-D3 sequence revealed it has 91.01% identity with the SSU rDNA of *S. hyrcania* (KT354242). The identity value with other sequences was all less than 91%.

A number of 38 ektaphelenchid+seinurid, a *Noctuidonema* sp., an *Anomyctus* sp., an *Peraphelenchus* sp., two aphelenchid and three rhabditid SSU sequences were selected for the SSU phylogeny. A number of 52 ektaphelenchid + seinurid, a *Noctuidonema* sp., two aphelenchid and three rhabditid LSU D2-D3 sequences were selected for the LSU phylogeny. The selection of the sequences for both analyses was based on the previous study of Pedram (2019) and close phylogenetic affinity of the ingroup taxa.

**Figure 3:**
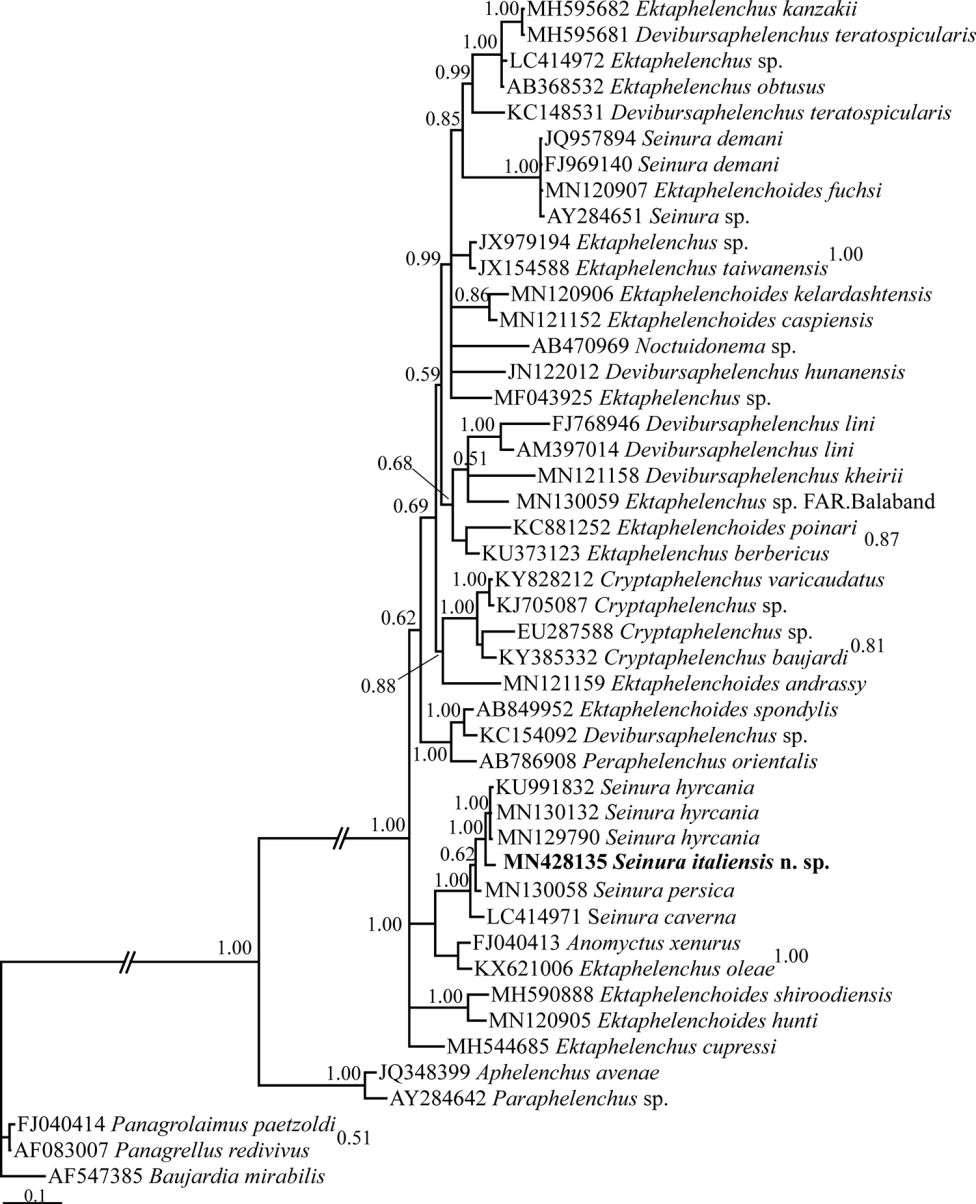
Phylogenetic relationships of *Seinura italiensis* n. sp. as inferred from Bayesian analysis using the near full-length SSU rDNA under GTR+I+G model. The Bayesian posterior probability (BPP) values more than 50% are given for appropriate clades. New sequence is in bold font.

The SSU dataset (Fig. 3) was composed of 1,622 characters of which 740 characters were variable. In this tree, the major clade including ektaphelenchids, seinurids, a *Noctuidonema* sp., a *Peraphelenchus* sp. and an *Anomyctus* sp. has received the maximal BPP. The four *Seiunra* spp. (*S. caverna*, *S. hyrcania*, *S. persica* and *S. italiensis* n. sp.) formed a maximally supported clade. The clade including *Ektaphelenchus oleae* (Miraeiz et al., 2017) + *Anomyctus xenurus* (Allen, 1940) was the sister clade to the *Seinura* clade. *S. demani* (Goodey, 1928; 1960) occupied a distant placement related to the *Seinura* clade (also see Discussion).

**Figure 4:**
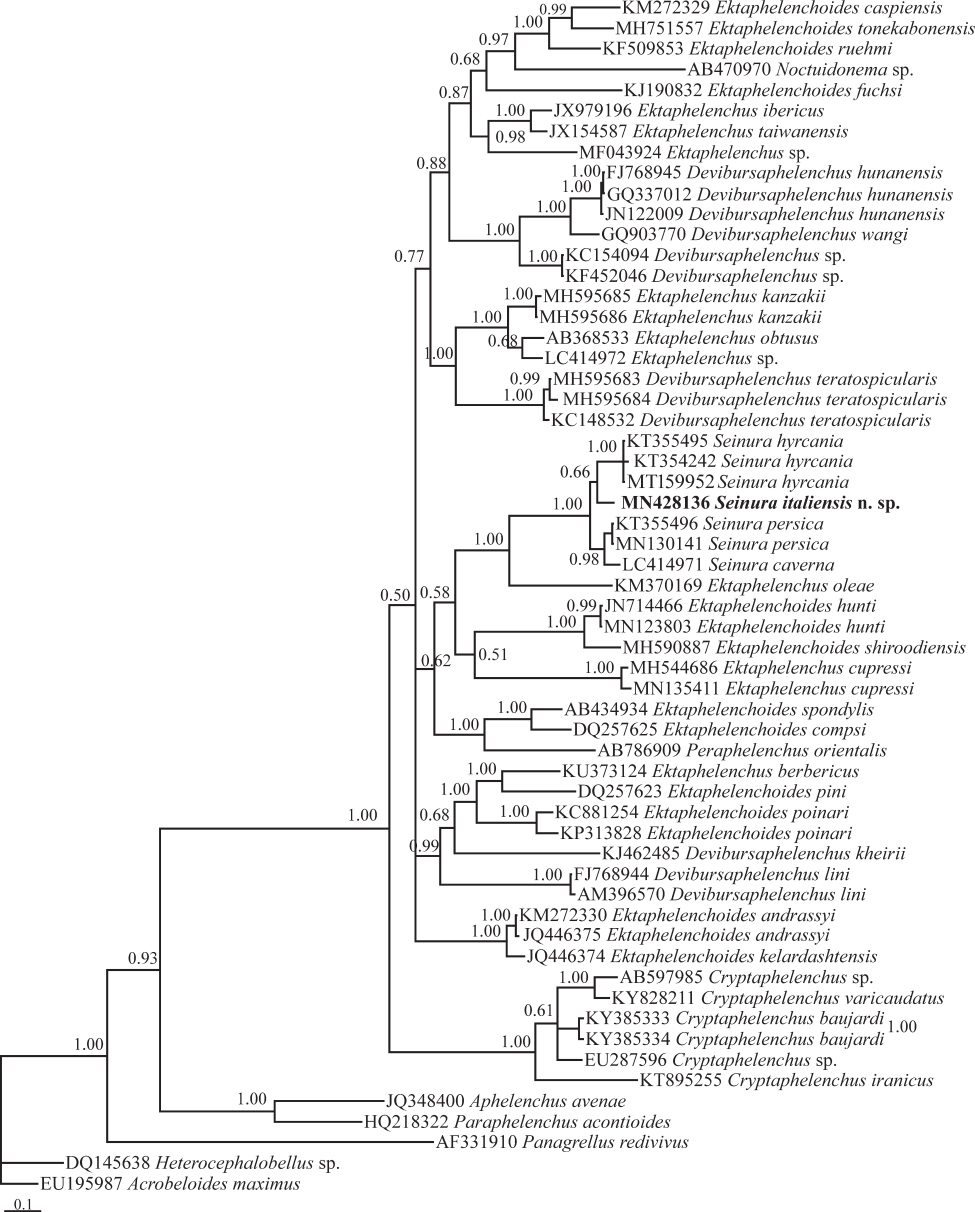
Phylogenetic relationships of *Seinura italiensis* n. sp. as inferred from Bayesian analysis using the near full-length LSU rDNA D2-D3 expansion segments under GTR+I+G model. The Bayesian posterior probability (BPP) values more than 50% are given for appropriate clades. New sequence is in bold font.

The LSU dataset (Fig. 4) was composed of 966 characters of which 672 characters were variable. In this tree, the major clade including ektaphelenchids, seinurids, a *Noctuidonema* sp. and a *Peraphelenchus* sp. has received the maximal BPP. Inside this major clade, the four *Seiunra* spp. (*S. caverna*, *S. hyrcania*, *S. persica*, and *S. italiensis* n. sp.) have formed a maximally supported clade. This clade is in a maximally supported sister relation with *Ektaphelenchus oleae*.

## Discussion

Based on our current knowledge, the *Seinura* species occupy a variety of habitats, the members of which were isolated from the rhizosphere of cultivated plants, animal manure, mushroom compost and bark and wood materials (Paesler, 1957; Goodey 1960; Massey 1971; Kaisa, 2000; Adeldoost et al., 2016; Kanzaki et al., 2018). The insect association of *Seinura* species is not well documented, and only *S. sutura* (Massey, 1971) and *S. arizonensis* (Massey, 1974) were known to be associated with bark beetles and weevils. A few other studies reported their presence in insect galleries of bark beetles (Kaisa, 2000; Chanu and Meitei, 2014; Bajaj, 2015); however, no insect association was reported.

Currently, the genus contains over 50 species (Kanzaki et al., 2018), and the literature review indicates that the majority of *Seinura* species have been reported from Asian (29 spp.), and others are reported from North American (11 spp.) and European continents (7 spp.). The low species reports from other continents may be a consequence of inadequate sampling or their excluding in identification programs.

In Seinurinae (Husain and Khan, 1967), the cephalic region is usually high, the stylet is long and slender, lacks basal knobs and the lumen is wide, median bulb is oblong, the prominent valve plates are situated in a post-median position; females have functional anus and elongate tail, usually more than four anal body widths long, often becoming attenuate or filiform. It could be separated from Ektaphelenchinae (Paramonov, 1964) by the females with functional vs vestigial to nonfunctional anus and rectum, separated from *Aphelenchoides* by the stylet (having a wide lumen, without basal knobs vs with small knobs and narrow lumen) and tail features (usually more than four anal body widths long, often becoming attenuate or filiform vs usually less than four anal body widths long, conoid, not filiform). The genus *Aprutides* (Scognamiglio et al., 1970) and *Papuaphelenchus* (Andrássy, 1973) also belong to Seinurinae, but the species are rare and characterized by their unique morphology. From the phylogenetic points of view, the genus *Seinura* is one of the genera with few recorded SSU and LSU sequences in GenBank database (e.g. in comparison with other aphelenchoidids like *Bursaphelenchus* spp., ektaphelenchids, etc.); thus, further data are necessary to infer a better phylogeny for it.

In our phylogenetic analyses, *Seinura* spp. formed a clade in both SSU and LSU phylogenies except *S. demani*, occupied a placement outside of the *Seinura* spp. clade. The latter species has a vestigial anus (Loof and Hooper, 1993) and most probably does not belong to *Seinura* and its taxonomic status and possible synonymy with *Ektaphelenchoides fuchsi* (Esmaeili et al., 2014) needs further study. The phylogenetic results presented during this study indicated the possible monophyly of *Seinura*; however, this needs further validations using molecular profiling of further species of the genus.

The predatory behavior of *Seinura* species seems to be the common feeding habit (Hunt, 1993; Kaisa, 2000; Adeldoost et al., 2016; Kanzaki et al., 2018). However, we do not know the feeding behavior of the new species as it was discovered under the quarantine inspections. The knowledge of its biology and ecology is an important issue to understand the evolutionary relationships among species and remains an open field for future research in case of the newly described species.
